# Antimicrobial resistance profile of staphylococcus *aureus* isolates isolated from ear discharges of patients at University of Hawassa comprehensive specialized hospital

**DOI:** 10.1186/s40360-017-0141-x

**Published:** 2017-05-15

**Authors:** Serawit Deyno, Alemayehu Toma, Mesfin Worku, Muluken Bekele

**Affiliations:** 1 Department of Pharmacology, School of Medicine, College of Medicine and Health Sciences, University of Hawassa, P. O. Box 1560, Hawassa, Ethiopia; 2 Department of medical laboratory technology, College of Medicine and Health Sciences, University of Hawassa, Hawassa, Ethiopia; 3 Department of ear, nose and throat, School of Medicine, College of Medicine and Health Sciences, University of Hawassa, Hawassa, Ethiopia

**Keywords:** Antimicrobial resistance, Staphylococcus aureus, Ethiopia

## Abstract

**Background:**

Drug-resistant microorganism are a growing global danger. Strains of *S. aureus* have developed resistance to many commonly used antimicrobials due to indiscriminate use of antimicrobials, and treatment becoming a challenge. Studies assessing pattern and determinants of S*. aureus* resistance in ear infection in Ethiopia are very scarce. This study presents overview of pattern and determinants S*. aureus* resistance from samples of ear discharge in Ethiopia.

**Method:**

A prospective cross-sectional study was conducted on patients who visit ENT clinic of University of Hawassa comprehensive specialized hospital from February 1, 2016 to November 1, 2016. 117 specimens were collected with sterile applicator cotton swab from 117 patients with ear discharge visiting the clinic. Data was fed and then edited, cleared, entered and analyzed using SPSS version 20.

**Result:**

The prevalence of *S.aureus infection* was 28.2%. *S. aureus* isolates revealed up to 97.0% level of resistance pattern to the antimicrobials tested. In the determination of the susceptibility of *S. aureus* on nine selected antibiotics by disk diffusion technique, 97.0% of the isolates were resistant to cloxacilin and 74.2% showed resistance to vancomycin. The overall rate of MDR was 100%, all of the isolates were found to be resistant to more than two tested antimicrobials.

**Conclusion:**

*S. aureus* has gotten frighteningly resistant to many of common antimicrobials. Resistance rate to vancomyin is terrifyingly high. It urges us to take measures to delay resistance. Emergence of resistance highlights the value of prudent prescribing of antimicrobials and avoiding their irrational use. Further researches focusing on identifying dynamics promoting resistance, identifying high risk strains and molecular basis of resistance are required.

## Background

Drug-resistant microorganisms are a growing danger to the global society. They endanger people in prosperous societies to poor nations. Antimicrobial resistance (AMR) is a long-standing problem with magnitude and speed it spreads becoming global most serious current public health problem [1–3].

The human Kind was overwhelmed by infectious diseases until the discovery of antimicrobials in the middle of twentieth century. Owing to discovery of various antimicrobial agents the ability to manage infectious diseases has greatly improved [4]. However, the beginning of the era of AMR were recorded soon after the discovery of penicillin, in which a number of treatment failures and occurrence of some bacteria no longer sensitive to penicillin started being noticed [5].

Microbial infection involving microorganisms poses a very serious public health problem all over the world especially in resource limited African countries. Among the many bacterial infections, *S. aureus* is the leading cause of nosocomial infections by gram-positive bacteria[6]. It is notoriously resistant to penicillin and many other antimicrobials [7].

Staphylococci resides with skin, glands and mucous membranes of almost all the warm blooded animals [8]. They prefer aerobic environment but can also grow in the absence of oxygen; range of temperature for growth is 6-44 °C (optimum 37 °C) and the range of pH is 4.2-9.3 (optimum 7). To date, there are 32 species and eight sub-species in the genus Staphylococcus, many of which preferentially colonize the human body, however, *S. aureus* and *S. epidermidis* are the two most characterized and studied strains [9, 10].

Strains of *S.aureus* have developed resistance to many commonly used antimicrobial due to indiscriminate use. Staphylococcal resistance to penicillin is mediated by β-lactamase production. First report of a penicillin-resistant strain of *S. aureus* was published in 1945, revealing its association with β-lactamase enzyme produced by the bacteria. The methicillin resistant *staphylococcus aureus* (MRSA) is a specific strain of the *S. aureus* bacterium that has developed antimicrobial resistance to all penicillin‘s, including methicillin and other narrow-spectrum β-lactamase-resistant penicillin antimicrobials [11].

Soon after methicillin was introduced into human medicine MRSA was observed [12]. MRSA has since emerged as a serious concern in human medicine. Although these organisms cause the same types of infections as other *S. aureus*, hospital associated strains have become resistant to most common antimicrobials, making treatment a great challenge.

Ear infection is a common clinical problem throughout the world and the major cause of preventable hearing loss in the developing world [13]. Microbial agents can infect the middle and external parts of the ear and may involve the skin, cartilage, periosteum, ear canal, and tympanic and mastoid cavities [14].About 65–330 million people suffer from ear infection worldwide and 60% of them had significant hearing loss [15]. The health and economic burden of ear infection is severe especially in Africa and other developing countries where the disease prevalence is high [16]. According to WHO report, Ethiopia belongs among the high ear infection burden countries [16].

It is a common tradition in Ethiopia that antibiotics can be obtained without prescription. This misuse of antibiotics by the public contributes to the emergence and spread of AMR rendering ear infection untreatable [17]. However, studies assessing pattern and determinant of S*. aureus* resistance in ear infection in Ethiopia are very scarce. This study presents the overview of pattern and determinants S*. aureus* resistance from samples of ear discharge in Ethiopia.

## Methods

Study setting and study period: This study was conducted at University of Hawassa Comprehensive Specialized Hospital from February 1, 2016 to November 1, 2016.

Study population and Study design: The source population was patients visiting ear, nose, and throat (ENT) clinic at University of Hawassa Comprehensive Specialized Hospital. The study population was patients who had suspected ear infection in Ear, Nose and Throat (ENT) clinic with clinical discharge. Hospital based cross sectional study design was implemented for this study.

Sampling technique: Convenient sampling technique was implemented in which patients who had developed ear infection and came to ENT outpatient department with clinically approved ear discharge during the study period were enrolled.

Inclusion and exclusion criteria: Patients who give their consent and had ear discharge with ENT specialist physician diagnosis was included in the study while patients who refuse to participate was excluded from the study.

Study variables: Socio-demographic, age, sex, previous infection, previous treatment history were considered as independent variables while growth of staphylococcus aureus isolates and antimicrobial susceptibility pattern (susceptible, resistance, intermediate) were considered dependent variables in this study.

### Broth preparation

Tryptone soya broth was used as a general purpose media. It was prepared according to the manufacturer‘s guideline. It was mixed until it completely dissolves on a hot plate and 5 ml was dispensed into each test tube and autoclaved at 120 0C for 15 minutes.

### Sample collection

Ear swab samples were collected using a sterile swab stick from patients with ear infections at University of Hawassa Comprehensive Specialized Hospital ENT clinic from February 1, 2016 to November 1, 2016. The ear swab sample was sent to the teaching laboratory of Hawassa University Comprehensive Specialized Hospital by Amies transport media (Oxoid Company) for immediate processing. Upon receipt sample was inoculated on Manitol Salt agar and Blood agar (Oxoid Company) plates and incubated at 37 °C aerobically. The swab was smeared on slide and then gram stained and examined microscopically.

### Characterization of isolated bacteria

After 24 hour incubation, bacteria isolates were characterized based on colonial appearance on Manitol salt agar and blood agar media. Gram stain was done to determine Gram reaction and bacterial morphology. After gram staining those colony shown to be gram positive cocci were subjected to further biochemical tests. Then the grown colony on primary culture media was subcultured on to nutrient agar to get pure colony which is suitable for biochemical test. Biochemical tests carried out were catalase and coagulase tests. Those catalase enzyme negative were considered as coagulase negative *Staphylococci* and coagulase test positive were considered as *S. aureus*

### Antimicrobial sensitivity testing

Antibacterial susceptibility testing was performed for all isolates according to the criteria of the National Committee for clinical Laboratory Standard (NCCLs) by disc diffusion method[18]. From a pure culture 3–5 selected colonies of bacteria were taken and transferred to a tube containing 5 ml nutrient broth and mixed gently until a homogenous suspension was formed and incubated at 37 °C until the turbidity of the suspension became adjusted to a McFarnald 0.5. A sterile cotton swab was used and the excess suspension was removed by gentile rotation of the swab against the inside surface of tube. The swab was then used to distribute the bacteria evenly over the entire surface of Mullen-Hinton agar. The inoculated plates were left at room temperature to dry for 3–5 minutes and a set of antibiotic discs such as cloxacillin (CLX) (5), cephalothin (CF) (30), vancomycin (30), amoxicillin-clavulanic acid (AMC) (30), chloramphenicol (C) (30 μg), amikacin (AN) (30), Kanamycin (30), Gentamycin (10), and Amoxicillin (2) were dispensed. The inoculated plate then incubated at 37oC for 24 hrs.

#### Data analysis

Data was edited, cleaned, entered and analyzed using SPSS version 20. Descriptive analysis such as frequencies and mean was used. Multiple antibiotic resistance (MAR) index was determined by following the procedure described by Krumperman [19]. A MAR index for an isolate is calculated as: number of antibiotics to which isolate is resistant/ total number of antibiotics against which isolate was tested. *P*-value of < 0.05 was considered statistically significant. The results were presented using tables.

#### Quality control

The quality of the data was maintained by strictly implementing quality control measures throughout the whole process of the laboratory work. Staining reagents, culture media and antibiotic discs was checked for their normal shelf life before use. All culture plates was stored at recommended refrigeration temperature (2–8 °C) after prepared and sterilized by autoclaving at 121 °C for 15 minutes. Antibiotic discs was stored at the same temperature range before use. All laboratory procedures was conducted based on recommended standard laboratory procedures. Pre-analytical, analytical and post-analytical stages of quality assurance are strictly followed in accordance to standard operating procedures of the microbiology research laboratory.

## Results

A total of 117 samples were collected from 117 patients with ear discharge. About 56.4% of the study participant were males. The mean age (SE) of the study participants were 20.4(1.3). The prevalence of *S.aureus* in our study was 33 (28.2%), in males 21(31.8%) and females 12(24.5%). It is found that growth of *S.aureus* in our sample has not showed association with any of socio-demographic and clinical characteristic variables tested in Table 1.

**Table 1:** Socio-demographic characteristics and clinical characteristics of patients with ear discharge at University of Hawassa Comprehensive Specialized Hospital, Hawassa, Ethiopia, February 1, 2016 to November 1, 2016

Variables		Growth of S. aureus		Total N (%)	*P*-value
		Yes (%)	No (%)		
Age	Under 5	7(41.2)	10(58.8)	17	ref
	6-15	10(31.2)	22(68.8)	32	0.6
	16 -30	13(27.1)	35(72.9)	48	0.6
	Above 31	4(20.0)	16(80.0)	20	0.2
Sex	Male	21 (31.8)	45 (68.2)	66	0.41
	Female	12 (24.5)	37 (75.5)	49	
History of previous infection	Yes	25 (28.4)	63(71.6%)	88	0.98
	No	6(28.6%)	15(71.4%)	21	
Previous treatment with antimicrobials	Yes	21(29.6)	50 (70.4%)	71	0.88
	No	11(28.2%)	28 (71.8%)	39	
Is there ear pain	Yes	26 (27.9)	67 (72.1%)	93	0.98
	No	9 (40.9%)	13 (59.1%)	22	
Is there itching of external ear	Yes	21(30.4%)	48(69.6%)	69	0.20
	No	5(17.9%)	23 (82.1%)	28	
Is there hearing problem	Yes	14(26.4%)	39(73.6%)	53	0.71
	No	14 (29.8)	33 (70.2)	47	
Is there fever	Yes	8(25%)	24(75%)	32	0.58
	No	20(30.3%)	46(69.7)	66	

Overall, *S. aureus* isolates revealed 3.0 – 97.0% level of resistance pattern to the antimicrobials tested. In the determination of the susceptibility of *S. aureus* on nine selected antibiotics by disk diffusion technique, 30(96.8%) of the isolates were resistant to Cloxacilin and 23(74.2%) showed resistance to vancomycin (Table 2). A lower level of resistance was observed to cefepime and amikacin.

**Table 2:** Antimicrobial resistance patterns of *s. aureus* isolates (*n* = 33) from ear discharges of study participants, at University of Hawassa comprehensive specialized hospital February 1, 2016 to November 1, 2016

	Antimicrobial agents	Sensitive N (%)	Intermediate N (%)	Resistant N (%)
1	Amoxicillin-clavulanic acid	13(39.4)	4(12.0)	16(48.5)
2	Cefepime	26(78.8)	5(15.2)	1(3.0)
3	Gentamycin	26(78.8)	1(3.0)	6(18.2)
4	Amikacin	32(97.0)	0	1(3.0)
5	Vancomycin	8(24.2)	2(6.1)	23(74.2)
6	Cloxacilin/Methicillin	1(3.0)	0	30(97.0)
7	Kanamycin	29(87.9)	0	4(12.1)
8	Cephalothin	27(81.8)	0	6(18.2)
9	Amoxicillin	3(9.1)	0	30(90.9)

Multi-drug resistance in this study was taken as resistance to more than one of the antimicrobial drugs tested. Multidrug-resistant (MDR) status of S. aureus isolates was tested against nine classes of antimicrobials. Accordingly, the overall rate of MDR was 100%, all of the isolates was found to be resistant to more than two tested antimicrobials. All of tested S. aureus showed multidrug-resistance shown by MAR index (0.22 to 0.66) in Table 3.

**Table 3:** Multidrug resistance profiles of *S. aureus* isolates to the tested antibiotics (*n* = 9) between February 1, 2016 to November 1, 2016 in University of Hawassa comprehensive specialized hospital

	Parameter	Frequency	MAR index
1	R2 = Van, Amx	1	0.22
2	R2 = Amx, Cxc	3	0.22
3	R3 = Gen, Amx, Cxc	2	0.33
4	R3 = Van, Amx, Cxc	5	0.33
5	R3 = Aug, Van, Amx	1	0.33
6	R3 = Aug, Amx, Cxc	1	0.33
7	R3 = Amx,Cxc,Clt	1	0.33
8	R4 = Aug, Van, Cxc,Amx	2	0.44
9	R4 = Van,Amx, Kan,Cxc	2	0.44
10	R4 = Van, Amx, Cxc, CLT	1	0.44
11	R4 = Van,Amx, Gen, Cxc	1	0.44
12	R4 = Aug, Van, Amx, Cxc	6	0.44
13	R5 = Aug,Van,Amx,Cxc,Clt	3	0.55
14	R6 = Aug, Van, Amx, Kan,Cxc, Gen	2	0.66
15	R6 = Van, Amx, Kan, Cxc, Clt, Gen	1	0.66
16	R6 = Aug,Van,Gen,Cxc,Kan,Amx	1	0.66

*Aug = amoxacilin-clavulanic acid, Van = Vancomycin, Amx = Amoxacilin, Cxc = cloxacilin, Kan = Kanamycin, Gen = gentamycin, Clt = Cephallothin, Cfp = cefepime, Amk = Amikacin

*MAR index = multiple antibiotic resistance index = no. of antimicrobials to which the isolate is resistant/no. of antibiotics to which the isolate is subjected

## Discussions

In this study, the prevalence of *S. aureus* was found to be 28.2% which is similar to studies conducted in many different parts of Ethiopia ranging from 24–28 [20–24].In this study, we estimated prevalence of *S. aureus* resistance to nine different antimicrobial agents commonly used in Ethiopia. Overall, the study provided evidence regarding the prevalence of *S. aureus* resistance to different antimicrobial agents based on ear discharge specimens collected from 117 patients.

It was found that *S. aureus* resistance to commonly available antimicrobial agents in Ethiopia was alarmingly high. The resistance to most preferred agents to *S.auerus* infection like methicillin and Vancomyicn have dangerously increased compared to previous similar studies [20–22, 25, 26]. About 96.8% of *S.aureus* was cloxacillin/methicillin resistant (MRSA) and 74.2%was vancomycin resistant (VRSA). VRSA rattles clinicians, since there is no drug of choice to treat life-threatening infections with MRSA.

The implication of high prevalence of MRSA and resistance to vancomycin, urges for better second-line drugs for suspected or verified *S. aureus* infections and surgical prophylaxis. Second-line drugs are more expensive and, because of their adverse effects, monitoring during treatment is advisable which increases the costs even further.

*S. aureus* resistance particularly MRSA in this study is higher than many of similar studies conducted in Ethiopia [17, 20, 23]. This indicates a rapid rise in MRSA. The WHO report shows MRSA prevalence between 33%-95% in Africa, 43%-45% in America, 13%-18% in Eastern Mediterranean Region, 27%-50% in Europe, 2%-80% in the South-East Asia region, and 4%-84% in the Western Pacific Region [27]. Our finding is much higher than the WHO report and calls greater attention to the threat.

High resistance suggests that the origin of the isolates were from highly resistant source where antimicrobials are often used. Global pattern of AMR shows variation among different geographic, socioeconomic strata and studies [27–29]. Variation may be to differences in time, place, design, and population involved in the study. Variation may be due to healthcare facilities conditions like implementation and monitoring of infection prevention policies and rationale antimicrobial usage which varies in different facilities. Studies are often conducted within a specified time and locality. It is reasonable to assume population under study might be infected by the same strains of the agent at specified period of time and location.

This study revealed aminoglycosides (amikacin and kanamycin) and fourth generation cephalosporin cefepime showed lower level of resistance. This may be due to rare use/availability of aminoglycosides (amikacin and kanamycin) in the treatment of infections in Ethiopia. Although other aminoglycosides (gentamycin and streptomycin) commonly available in Ethiopia. common use of gentamycin and streptomycin may not contribute to emergence of resistance to amikacin and kanamycin due to low cross-resistance among aminoglycosides [30]. Cefepime is totally absent in Ethiopia. It appears that amikacin, kanamycin and cefepime more effective antimicrobials in the treatment of *S. aureus* infections in Ethiopia

Many factors contribute to AMR. First, lack of infection prevention contributes to recurrent infection then to spread of resistant strains. Second, misuse of antimicrobials from prescription–dispensing-to patient use [31]. In Ethiopia, it is a common practice that antibiotics can be purchased without prescription, which leads to misuse of antibiotics by the public [32]. Third factor could be misuse of antibiotics by health professionals and non-standardized practice [31]. The fourth factor could be poor hospital hygienic conditions [33].

Lack of culture and routine antimicrobial susceptibility testing which diverts to empiric therapy may also contribute to the spread [28]. In line to strategies for prevention and containment of *S. aureus* there is a need for innovative way of halting AMR. Combination therapy, design of new drugs and availability of alternative antimicrobial agents will play important role in fighting against AMR to *S. aureus*.

Multi-drug resistant S*. aureus* isolates to antimicrobials was alarmingly high in this study in which resistance up to six drug was recorded. Empirical prophylaxis and treatment needs careful selection of effective drugs. It needs continuous monitoring of the antimicrobial susceptibility surveillance system of *s. aureus* for the selection of appropriate therapy, developing the antimicrobials policy and limiting the use of reserve antibiotics. Strict consideration for *s. aureus* infection and proper usage of antimicrobials policy are recommended in decreasing the incidence and occurrence of multidrug resistant *s. aureus* infections.

The interpretation of this findings requires considering the limitations thereof. First, this is invitro testing and may not equate to patient outcome. Second, identification of different resistant genes like mecA, mecC and PVL-toxin which provides insight into the distribution of strains and the extent of antimicrobials susceptibility patterns in the area has not been included. Third, the small sample size of the current study may also be a limitation.

## Conclusions

This study reveals that *S. aureus* has gotten frighteningly resistant to many of common antimicrobials used in Ethiopia. It is highly resistant to amoxicillin, vancomycin, amoxicillin -clavulanic acid, Gentamycin, kanamycin, amikacin. Resistance to vancomyin is terrifyingly high. It urges us to include novel drugs in national drug list in Ethiopia in order to delay resistance and treat resistant strains. Emergence of VRSA highlights the value of prudent prescribing of antimicrobials (including vancomycin) and avoiding their irrational use. Further researches which focus on identifying dynamics promoting resistance, identifying high risk strains and molecular genetic basis of resistance are needed.

## Abbreviations

AMR: Antimicrobial resistance
ENT: Ear nose throat
MAR: multiple antibiotic resistance
MRSA: Methicillin resistant *Staphylococcus aureus*
NCCLs: National Committee for clinical Laboratory Standard
*S. aureus*: *Staphylococcus aureus*
VRSA: Vancomycin resistant *Staphylococcus aureus*
